# Case report: A case of sepsis caused by rickettsial infection-induced hemophagocytic syndrome

**DOI:** 10.3389/fmed.2023.1209174

**Published:** 2023-08-07

**Authors:** Yanli Cao, Peijun Liu, Qiuling Song, Jing Wang

**Affiliations:** Department of Respiratory and Critical Care Medicine, The Central Hospital of Enshi Tujia and Miao Autonomous Prefecture, Enshi, China

**Keywords:** Rickettsia, sepsis, multiple organ dysfunction, hemophagocytic syndrome, immune imbalance

## Abstract

Hemophagocytic lymphohistiocytosis (HLH) is a rare histiocytic disorder characterized by reactive hyperplasia of the mononuclear phagocytic system, which is primarily caused by dysfunction of cytotoxic killer cells and natural killer cells, leading to antigen clearance barriers and the overactivation of the mononuclear phagocytic system due to continuous antigen stimulation. HLH encompasses a group of clinical syndromes marked by the overproduction of inflammatory cytokines. A 68-year-old Chinese man presented with persistent fever, chills, nausea, and vomiting; the patient had no history of any underlying conditions. Laboratory investigations revealed decreased levels of red blood cells, white blood cells, and platelets, along with reduced natural killer cell activity, increased CD25, hyperferritinemia, and the detection of Rickettsia DNA in his blood, meeting the diagnostic criteria of the Histiocyte Society HLH-2004 guidelines. The patient was treated with antibiotics, improving anemia, glucocorticoid therapy, and continuous renal replacement therapy (CRRT), temporarily improving his condition. However, the patient died after 2 years from chronic renal failure caused by septic shock.

## Introduction

Rickettsiosis, a condition driven by transmission of intracellular gram-negative bacteria, is further categorized as spotted fever or typhus, generally resulting from arthropod infection in human populations (1, 2). Hemophagocytic lymphohistiocytosis (HLH), a rare and potentially fatal immune syndrome, is characterized by uncontrollable activation of cytotoxic lymphocytes and macrophages, leading to cytokine-mediated tissue damage and subsequent organ failure (3). Clinical and laboratory observations of HLH include fever, splenomegaly, organ failure, cytopenias, hypertriglyceridemia, hyperferritinemia, hemophagocytosis, and decreased NK cell activity (4, 5). Currently, HLH associated with rickettsial sepsis is an infrequent event. This article presents a unique case study for critical care and hematology specialists.

## Case report

A 68-year-old Chinese male presented with a fever, with a temperature of up to 39.5°C, and continued to experience chills for more than 7 days. His red blood cell count (RBC), hemoglobin (Hgb), and platelet (Plt) count were all reduced (Table 1). He was admitted to the intensive care unit of Enshi Central Hospital in Hubei province. After the onset of the disease, the patient underwent antibiotic therapy at a local clinic, receiving a daily intravenous dose of 0.5 g levofloxacin for over 7 days. However, there was no significant improvement in the patient's condition. The patient was a farmer who lived in a karst mountain forest environment. Before this, he was in good health and had no other chronic diseases.

**Table 1:** Laboratory result at admission.

**Project**	**On admission**	**Before discharge**	**Reference range**
White blood cell	2.39 × 10^9^/L	3.31 × 10^9^/L	4~10 × 109/L
Neutrophil percentage	87.60%	48.90%	50~70%
Lymphocyte	0.18 × 10^9^/L	1.19 × 10^9^/L	1~4 × 109/L
Monocyte	0.11 × 10^9^/L	0.4 × 10^9^/L	0.26~0.8 × 10^9^/L
Red blood cell	1.91 × 10^12^/L	2.60 × 10^9^/L	4~5.5 × 10^9^/L
Hemoglobin	58 g/L	79 g/L	120~160 g/L
Hematocrit	0.184	0.248	0.41~0.49
Platelet	37 × 10^9^/L	84 × 10^9^/L	90~300 × 10^9^/L
ALT	112 U/L	30 U/L	5~40 U/L
AST	126 U/L	32 U/L	5~40 U/L
Total bilirubin	9.5 umol/L	20 umol/L	2~20 umol/L
Total protein	52.98 g/L	61.50 g/L	60~82 g/L
Albumin	27.39 g/L	35.51 g/L	33~48 g/L
Globulin	25.59 g/L	25.99 g/L	18~35 g/L
Lactic dehydrogenase	332 U/L	155 U/L	109~240 U/L
Procalcitonin	58.05 ng/ml	0.36 ng/ml	0~0.5 ng/ml
C-reactive protein	122.81 mg/L	6.27 mg/L	0~8 mg/L
Triglyceride	2.05 mmol/L	1.83 mmol/L	0.45~1.7 mmol/L
Prothrombin time	12.2 s	13.0 s	9.4~12.5 s
D-dimer levels	5.613 ug/ml	1.824 ug/ml	0~0.243 ug/ml
FDP	30.896 ug/ml	14.542 ug/ml	0~2.01 ug/ml
Fibrinogen	3.17 g/L	2.52 g/L	2.38~4.98 g/L

In the initial physical examination, the patient's body temperature was 36.3°C, pulse rate was 88 beats per minute, breathing rate was 18 breaths per minute, blood pressure was 100/60 mmHg, and both breathing and heart rate were regular. The patient exhibited a lean physique and a notably pale skin complexion while maintaining mental clarity. There was no rash or bleeding point all over his body. A scar of size 0.50^*^0.5 cm^2^ was visible in the lower right abdomen, but there were no other apparent abnormalities.

Preliminary laboratory examination showed a significant decrease in white blood cells, lymphocytes, monocytes, red blood cells, hematocrit, platelets, and platelet volume. The neutrophil ratio was increased. Serum levels of alanine aminotransferase (ALT), aspartate aminotransferase (AST), alkaline phosphatase (ALP), lactate dehydrogenase (LDH), C-reactive protein (CRP), and procalcitonin (PCT) were significantly elevated. In addition, prothrombin time (PT) was increased, and D-dimer levels and fibrinogen degradation products (FDPs) were elevated (Table 1). All tests for viral respiratory pathogens, typical nuclear antibody spectrum, human immunodeficiency virus, and syphilis were negative. The patient was subjected to three distinct blood cell culture analyses, and all conclusively reported negative results. Concurrently, serological testing for antibodies associated with the Epstein–Barr Virus (EBV) and the Cytomegalovirus (CMV) was conducted, revealing negative outcomes. The nasal swab tested negative for the presence of COVID-19 RNA. Ferritin levels were extremely high, and the patient tested positive for hepatitis B virus antigen. Natural killer cell activity was 3.75%, and the activity was decreased. All inflammation markers associated with HLH were elevated, including CD25 (3,644 U/ml), ferritin (above 1,500 ng/ml, reference range: 23.9~336.2 ng/ml), interleukin-6 (6.49 pg/L), and TNF-1(19 fmol/ml). Through high-throughput gene detection (NGS) of blood PMseq-DNA, we identified hepatitis B virus (HBV) and rickettsia presence in the samples (Supplementary Table S1, Supplementary Figure S1). The results of the HBV DNA were 9.35^*^10^4^ IU/ml. A regimen of 0.25 mg/day entecavir was used to treat patients with chronic hepatitis B.

**Figure 1:**
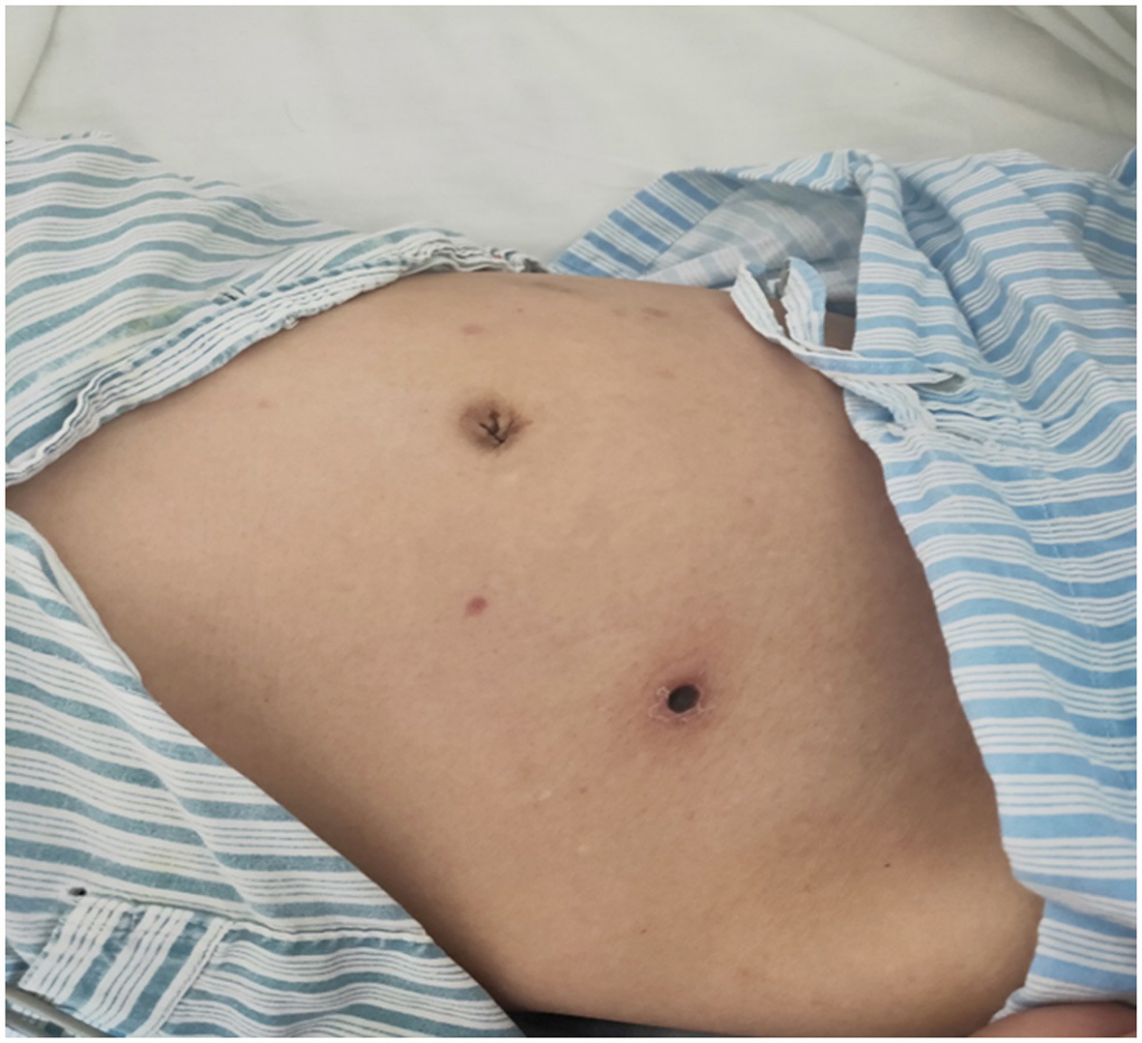
Scar from a tick bite on the patient's lower right abdomen.

**Figure 2:**
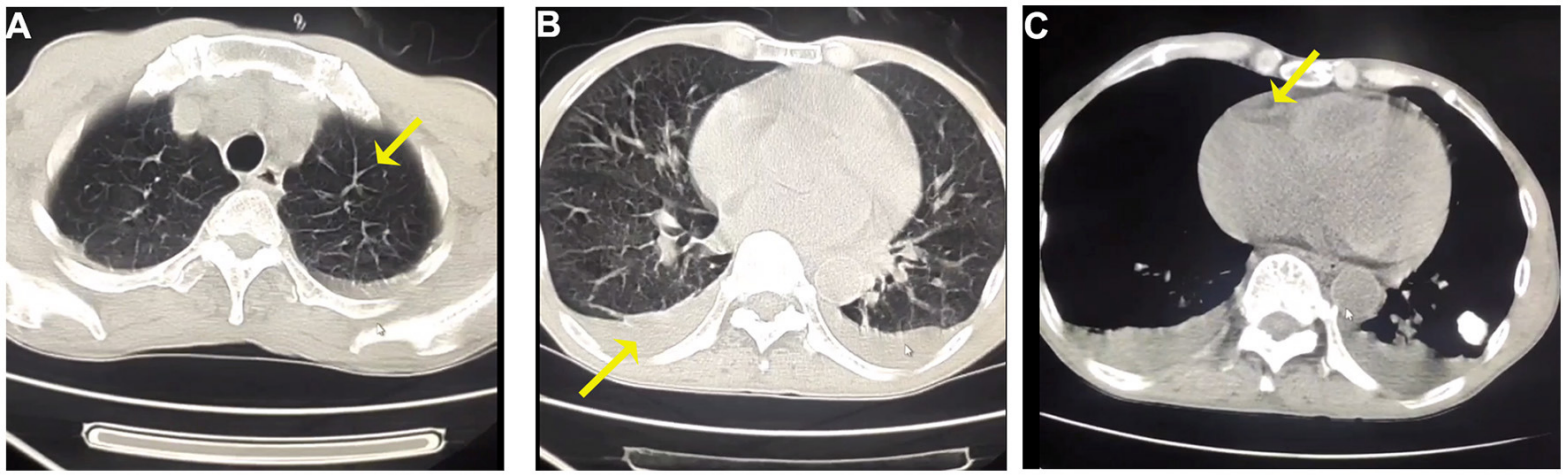
**(A)** Representative CT images of interlobular septal thickening at chest CT scan of both upper lungs. **(B)** Bilateral pleural effusions in the lower lungs. **(C)** The mediastinal window of the chest CT reveals a small amount of pericardial effusion.

**Figure 3:**
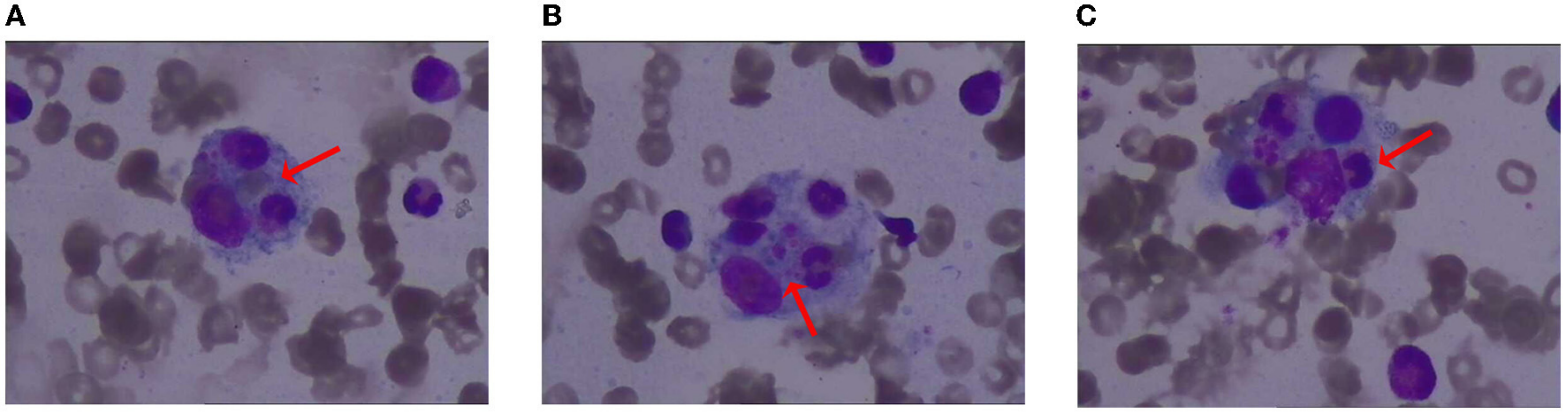
Illustration of bone marrow staining. **(A)** Erythrocyte phagocytosis; **(B)** platelet phagocytosis; **(C)** granulocyte phagocytosis.

The patient has a 0.5 × 0.5 cm^2^ scar on his lower right abdomen, caused by a mosquito bite that occurred more than 10 days before hospital admission (Figure 1). The patient underwent cardiac and abdominal ultrasound imaging, which indicated right heart enlargement—the right atrium and ventricle transverse diameters were 5.1 cm and 4.8 cm, respectively. The left ventricular ejection fraction was 71%, and the left ventricle's short-axis fractional shortening was 40%. The liver was unremarkable, with clear intrahepatic vasculature. The spleen was normal, measuring 3.7 cm in thickness, with a smooth capsule and no splenic vein dilation. The renal parenchyma of both kidneys exhibited increased echogenicity, accompanied by the presence of small cysts. A chest CT scan revealed two inflammatory lung lesions and mild interstitial pulmonary edema (Figure 2A). Additionally, bilateral pleural effusion, lower pulmonary partial atelectasis, and bilateral pleural thickening were observed (Figure 2B). Aortic thickening was also observed, and some fluid was present in the pericardial cavity (Figure 2C). A peripheral blood smear was performed on the patient. The findings revealed that the white blood cell classification ratio and morphology were predominantly standard. There was variation in the size of red blood cells. Platelet distribution appeared satisfactory, and no blood parasites were observed (Supplementary Figure S2). The bone marrow puncture revealed significantly active proliferation of bone marrow nucleated cells, accompanied by erythrocyte hyperplasia and erythrocyte phagocytosis (Figure 3A). Most of the cells were granulomatous macrophages, and scattered platelet phagocytosis was also observed (Figure 3B). Granulocyte hyperplasia was characterized by immature neutrophils and band-shaped neutrophils (Figure 3C).

The patient presented with a diagnosis of HLH and multiple organ dysfunction syndrome (MODS), as he fulfilled seven out of the eight diagnostic criteria for HLH-2004 (6). These criteria include fever, peripheral blood cytopenia affecting at least three lineages, hypertriglyceridemia, the presence of hemophagocytic cells in the bone marrow without malignancy, decreased natural killer (NK) cell activity, hyperferritinemia, and CD25 levels above 2,400 U/ml. Furthermore, PMseq-DNA test results indicated the presence of septic shock caused by Rickettsia infection, a secondary complication of multiple organ dysfunction. Treatment consisted of fluid rehydration, blood transfusion, combination therapy with meropenem and minocycline, anti-inflammatory therapy with methylprednisolone sodium succinate, several sessions of renal replacement therapy (CRRT), and other supportive measures. The patient's condition improved over time, with the resolution of fever, and ultimately, he was discharged from the hospital.

## Discussion

The significance of HLH is particularly relevant in the pediatric setting, with adult mortality rates ranging between 20 and 88% (7). HLH is typically classified into two types: (1) primary or familial HLH and (2) secondary HLH (8). It is a potentially fatal illness commonly precipitated by infection, autoimmune disorders, or malignancy (9). In clinical terms, HLH shares similarities with bacterial sepsis or systemic inflammatory response syndrome, with inflammatory overactivity and pathophysiological characteristics closely related to septic shock. Furthermore, clinical and laboratory features of septic shock are indistinguishable (10, 11).

The patient had septic shock, characterized by microbial infection causing fever, leukocyte imbalances, and multiple organ dysfunction syndrome (MODS) (12). He presented with persistent high fever, liver and kidney insufficiency, and effusion in multiple serous cavities. The lack of adequate circulating blood volume was believed to cause MODS. Proper fluid management is crucial for resuscitation, as positive water balance is linked to more extensive organ failure and mortality (13). Our investigation revealed that the sepsis was caused by rickettsial infection, an intracellular, specialized gram-negative bacterium that can infect humans through arthropods such as ticks (14). A study by P. Aarthi et al. utilizing PCR-based DNA sequencing found that rickettsial infection could lead to neonatal sepsis (15). Tick-borne infections can cause a range of afflictions, including Rocky Mountain spotted fever, a severe tick-borne illness capable of inducing adult respiratory distress syndrome, septic shock, and myocarditis. Symptoms include elevated cardiac enzyme levels and declining myocardial function. However, the condition usually improves with antibiotic treatment (16).

A few reports of rickettsial infection causing hemophagocytic syndrome exist, though this condition occurs more frequently in children with an incomplete immune system (17, 18). The patient's condition must be distinguished from disorders such as thrombotic thrombocytopenic purpura (TTP) and hemolytic uremic syndrome (HUS). TTP is a rare thrombotic microvascular disease characterized by microvascular pathogenic hemolytic anemia (19). The patient had a trilineage decrease in red blood cells, white blood cells, and platelets. Significantly, there was no evidence of bleeding or clot development anywhere on the patient's body, prompting us to consider ruling out TTP from the differential diagnosis. HUS, predominantly observed in children, presents as a clinical triad of thrombocytopenia, anemia, and acute kidney injury. It is frequently associated with an *E. coli* infection (20). The patient did not exhibit symptoms such as diarrhea; thus, an intestinal disorder was not suspected. Considering the medical history, we can rule out the possibility of HUS. Despite the patient's slight rise in HBV DNA levels, an abdominal color Doppler ultrasonography scan clearly ruled out liver cirrhosis and liver injury. As a consequence, we ruled out the possibility of hepatitis B-related HLH.

Rickettsial infection is a zoonotic disease that is transmitted by arthropods. In this case, the patient resides in the mountains where arthropods are abundant. The diagnostic method for Rickettsia typically involves a combination of polymerase chain reaction (qPCR) and immunofluorescence detection. If collected early during the infection, specimens may not contain antibodies, but the probability of positive PCR increases (21). Rickettsial infection is usually treated with potent antibiotics such as doxycycline or minocycline. However, tigecycline has also been reported to treat Rickettsial meningitis since it increases drug concentrations in the blood (22). HLH is vulnerable to life-threatening complications such as multiple organ dysfunction syndrome (MODS) and disseminated intravascular coagulation (23). Immediate identification and intervention are crucial for improving patient prognosis. Treatment options for this condition vary, including immunosuppressive agents, cytotoxic chemotherapy, and hematopoietic stem cell transplantation (24).

Initially, we planned to provide chemotherapy to the patient after his condition had improved, but he declined and left the hospital. Following his discharge, we conducted numerous follow-ups and discovered that the patient had fever for 2 years. However, his kidney function and mild anemia remained problematic. Sadly, the patient died 2 years later due to persistent kidney failure.

## Conclusion

HLH is a rare yet potentially fatal disorder requiring high suspicion for prompt diagnosis and management. In the initial phases of secondary HLH, precise management of anti-infectives, shock prevention, and continuous renal replacement therapy have demonstrated positive effects of patient treatment. Unfortunately, limited access to treatment options due to financial constraints and concerns over potential side effects of subsequent drug therapies often deter patients from seeking further medical interventions. Despite refusing additional treatment, the patient exhibited prolonged survival.

## Data availability statement

The original contributions presented in the study are included in the article/Supplementary material, further inquiries can be directed to the corresponding author.

## Ethics statement

The studies involving human participants were reviewed and approved by Ethics Committee of Enshi Tujia and Miao Autonomous Prefecture Central Hospital. The patients/participants provided their written informed consent to participate in this study. Written informed consent was obtained from the individual(s) for the publication of any potentially identifiable images or data included in this article.

## Author contributions

YC contributed to the case collection, documentation, and writing of the manuscript. PL conducted extensive literature reviews and analysis of case studies. QS participated in image interpretation. JW contributed to the design aspect of the research and provided editorial support for the manuscript. All authors contributed to the article and approved the submitted version.
