# Supplementary material for: Case report: A case of sepsis caused by rickettsial infection-induced hemophagocytic syndrome
Source: Front Med (Lausanne). 2023 Aug 7;10:1209174. doi: 10.3389/fmed.2023.1209174 (PMC10440429; doi:10.3389/fmed.2023.1209174)
Supplement: Supplementary file 1 [file Table_1.doc]

**Table S1|** HlH-associated inflammatory molecules and PMseq-DNA

| Project | Reslults |
| --- | --- |
| Nk cells activity | 3.75% |
| CD25 | 3644 U/ml |
| [Ferritin](javascript:;) | ＞1500 ng/ml |
| Interleukin-6 | 6.49 pg/L |
| TNF-α1 | 19 fmol/ml |
| Hepatitis virus | 3 |
| Rickettsia | 44 |
