# Supplementary figures and images for: Case report: A case of sepsis caused by rickettsial infection-induced hemophagocytic syndrome
Source: Front Med (Lausanne). 2023 Aug 7;10:1209174. doi: 10.3389/fmed.2023.1209174 (PMC10440429; doi:10.3389/fmed.2023.1209174)

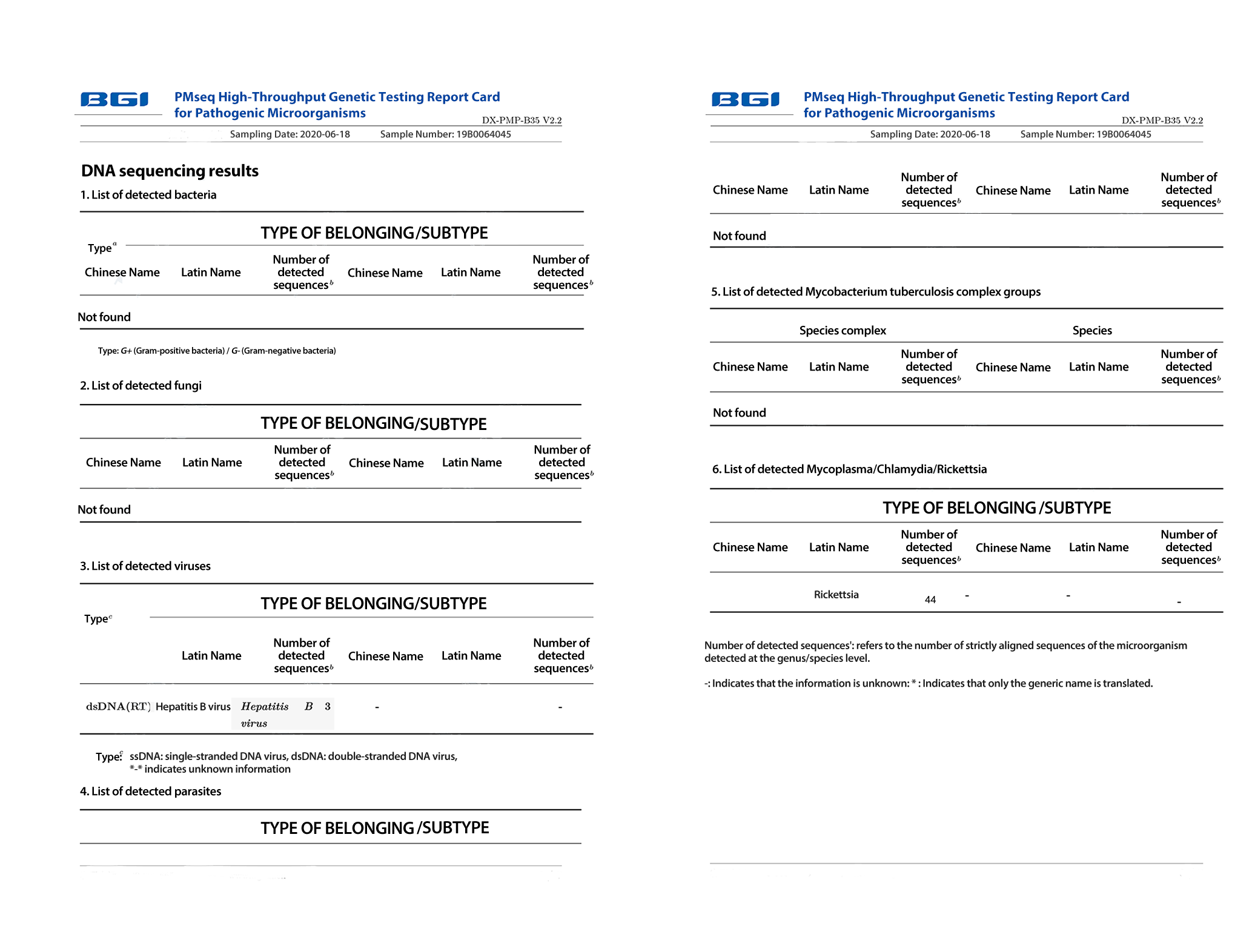

Supplement: Supplementary file 2 [file Image_1.tif]

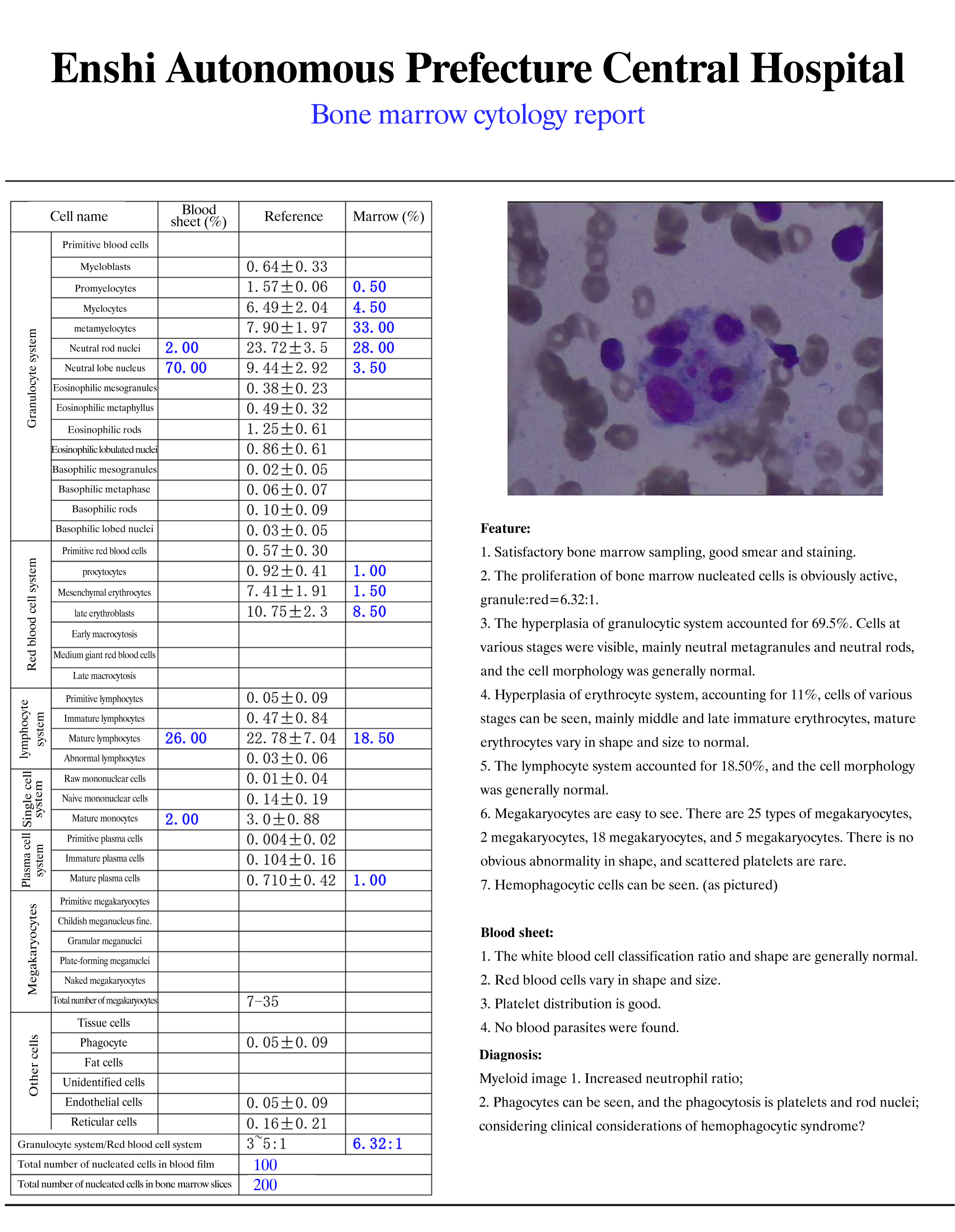

Supplement: Supplementary file 3 [file Image_2.tif]
